# Beneath the Surface: Exploring Hidden Threats of Long-Term Corticosteroid Therapy to Bone Density

**DOI:** 10.7759/cureus.55109

**Published:** 2024-02-27

**Authors:** Sultan A Alfaedi, Majd F Kubbara, Abdullah A Alaithan, Hamad M Alhudhaif, Ahmed A Al Abdullah, Hussain M Sahool, Mohammed S AL Jawad, Mohammed A Almatar, Ibrahim R Alnakhli, Mohammed A Altawili

**Affiliations:** 1 Orthopedic Surgery, King Abdallah Medical Complex, Jeddah, SAU; 2 General Practice, Maternity and Children's Hospital, Dammam, SAU; 3 General Practice, Ministry of Health, Riyadh, SAU; 4 General Practice, Omran General Hospital, Alahsa, SAU; 5 Orthopaedics, King Faisal University, Qatif, SAU; 6 General Practice, Qatif Central Hospital, Qatif, SAU; 7 Orthopedics Department, King Fahad Hospital, Jeddah, SAU; 8 General Practice, Maternity and Children Hospital, Medina , SAU; 9 General Practice, Alaziziyah Primary Health Care Center, Tabuk, SAU

**Keywords:** bone loss, bone density, bone mineral density, corticosteroid use, corticosteroid

## Abstract

Within the field of medical treatments, corticosteroids are potent substances that efficiently reduce inflammation and immunological responses, making them essential for the management of a wide range of medical ailments. However, continued use of these synthetic drugs presents a serious risk: the onset of osteoporosis brought on by corticosteroids.

Determining the complex pathways by which corticosteroids cause a general disturbance in bone metabolism, suppress osteoblast function, increase osteoclast activity, and upset the delicate balance of bone remodelling emphasizes the need for all-encompassing management and prevention approaches. In this review, we aim to expose the complexities of corticosteroid-induced bone loss and urge for personalized, proactive measures to improve long-term therapeutic outcomes.

## Introduction and background

Corticosteroids, often known as steroids, are a class of synthetic medications that replicate the effects of cortisol, a hormone generated naturally by the adrenal glands [1]. Because of their capacity to inhibit the immune system and reduce inflammation, these potent anti-inflammatory drugs are widely utilized in the treatment of a variety of medical problems [2]. Their uses range from treating autoimmune disorders and allergic reactions to respiratory ailments, skin issues, and certain forms of cancer. Corticosteroids produce their therapeutic effects by attaching to glucocorticoid receptors, modifying gene expression, and thwarting immunological responses, Prolonged use of corticosteroids, although beneficial in reducing inflammation and symptoms, is linked to numerous side effects, including serious implications for bone health [3,4].

A person's ability to support their body structurally, protect critical organs, and store minerals needed for a variety of physiological processes all depend on their ability to maintain healthy bones [5]. Bone remodelling, the process of maintaining healthy bones, depends on a fine balance between bone creation and resorption. While osteoclasts remove and break down old or damaged bone tissue, osteoblasts oversee the creation of new bone. A disturbance in this balance may result in ailments such as osteoporosis, which is marked by a reduction in bone mass and a heightened vulnerability to fractures [6,7]. Corticosteroids have varying effects on bone health depending on the patient and the ailment. This partnership is complicated for many reasons. A greater risk of bone loss is associated with higher doses and longer durations of corticosteroid medication, which are important factors to consider. Furthermore, an individual's sensitivity to corticosteroid-induced bone loss might be influenced by age, gender, nutritional status, and pre-existing bone problems [8,9]. Corticosteroids interfere with natural bone remodelling processes, which influences bone health. Extended use of corticosteroids has been associated with decreased osteoblast activity, increased osteoclast-mediated bone resorption, and decreased bone formation. This imbalance, which is known as corticosteroid-induced osteoporosis, causes bone loss and increases the risk of fractures [10,11]. Given the extensive and prolonged use of corticosteroids in a variety of medical disorders, the negative effects on bones are especially worrying. To control symptoms and stop the progression of chronic inflammatory diseases such as rheumatoid arthritis, asthma, or inflammatory bowel disease, patients frequently need long-term corticosteroid medication [12]. Beyond their apparent benefits, the shadows cast by these medications on bone health demand careful consideration, urging us to explore, analyze, and ultimately mitigate the obscured risks they harbour.

## Review

Mechanism of action of corticosteroids

Corticosteroids exert their therapeutic effects through a complex mechanism of action, primarily involving anti-inflammatory and immunosuppressive pathways. These synthetic hormones, which closely resemble cortisol, the body's natural stress hormone, interact with glucocorticoid receptors to modulate gene expression and influence various physiological processes [13].

Corticosteroids exert their anti-inflammatory effect by inhibiting proinflammatory genes. They enter cells and bind to glucocorticoid receptors located in the cytoplasm. This binding facilitates the translocation of the corticosteroid-receptor complex into the cell nucleus. Within the nucleus, the complex modulates gene expression by interacting with specific DNA sequences. Corticosteroids predominantly suppress the transcription of proinflammatory genes, such as those encoding cytokines (e.g., interleukins and tumour necrosis factor-alpha), chemokines, and adhesion molecules [14].

**Figure 1:**
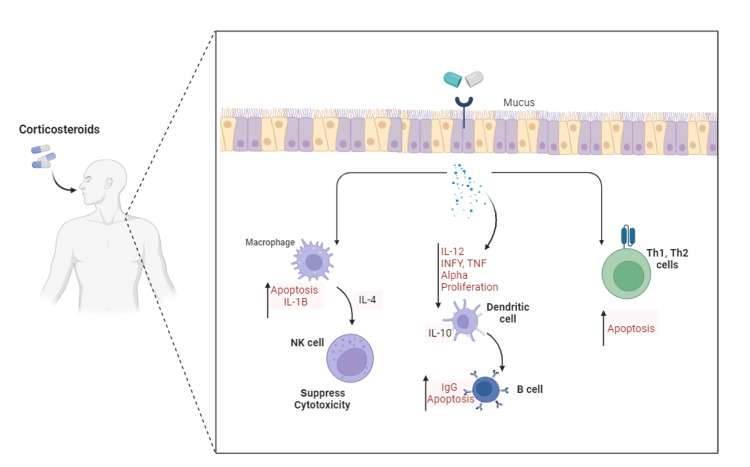
A summary of the inflammatory mechanism of corticosteroids Th: T helper cells, IL: interleukins; TNF: Tumor necrosis factor; INF: interferon. It is an original artwork designed by the authors.

Furthermore, corticosteroids interfere with the production of inflammatory mediators by inhibiting phospholipase A2, an enzyme involved in the synthesis of prostaglandins and leukotrienes. This inhibition results in a decreased release of arachidonic acid, the precursor of these inflammatory mediators [14,15]. Additionally, corticosteroids dampen the activation of immune cells, including T lymphocytes and macrophages. They inhibit the expression of major histocompatibility complex (MHC) molecules and downregulate co-stimulatory signals required for T-cell activation. By modulating immune cell activity, corticosteroids reduce the overall inflammatory response [14,15]. A summary of the inflammatory mechanism of corticosteroids is shown in Figure 1.

Corticosteroids induce apoptosis (programmed cell death) in lymphocytes, particularly T cells. This apoptotic effect reduces the number of circulating immune cells, leading to immunosuppression [16]. Corticosteroids suppress the production of various cytokines involved in the immune response. Interleukin-2 (IL-2), a key cytokine for T-cell proliferation, is significantly inhibited by corticosteroids [17]. Corticosteroids inhibit the migration of leukocytes to sites of inflammation by reducing the expression of adhesion molecules on endothelial cells. Corticosteroids can decrease the production of immunoglobulins, particularly IgG, by B cells [18,19].

Contribution to adverse effects on bone metabolism

While the anti-inflammatory and immunosuppressive actions of corticosteroids are crucial for managing various medical conditions, these mechanisms can contribute to adverse effects on bone metabolism. The delicate balance between bone formation and resorption, essential for maintaining bone density, is disrupted by corticosteroid therapy [20].

Osteoblasts are responsible for synthesizing new bone tissue. Corticosteroids can suppress osteoblast activity, leading to a reduction in bone formation. The inhibition of key osteogenic genes and proteins, such as runt-related transcription factor 2 (RUNX2) and osteocalcin, contributes to impaired osteoblast function [21]. Corticosteroids stimulate the differentiation and activity of osteoclasts, the cells responsible for bone resorption. Increased osteoclast activity results in the breakdown of bone tissue, leading to a net loss of bone mass. The net effect of corticosteroids on bone metabolism is an imbalance favouring bone resorption over formation. This imbalance results in a gradual reduction in bone mineral density (BMD), leading to conditions like corticosteroid-induced osteoporosis [7,22,23]. Corticosteroids can impair calcium absorption in the intestines and increase renal calcium excretion. This disruption in calcium homeostasis further contributes to bone demineralization. Corticosteroids may hinder the synthesis of collagen, a crucial component of the bone matrix. Impaired collagen formation can compromise the structural integrity of bone tissue [7,22,23].

Factors influencing corticosteroid-induced bone loss

Higher doses and longer durations of corticosteroid therapy are associated with an increased risk of bone loss. Short-term, high-dose corticosteroid bursts may have a lower impact on bone health compared to chronic, lower-dose regimens. Factors such as age, gender, genetics, and pre-existing bone conditions influence an individual's susceptibility to corticosteroid-induced bone loss. Older adults and postmenopausal women are generally at higher risk [24,25]. Certain medications, such as anticonvulsants and heparin, may interact with corticosteroids and exacerbate bone loss. Adequate calcium and vitamin D intake can mitigate the impact of corticosteroids on bone health [24,25].

Normal bone remodeling: balancing formation and resorption

Bone remodelling is a dynamic and continuous process crucial for maintaining bone strength, repairing micro-damage, and regulating mineral homeostasis. This tightly regulated cycle involves the coordinated activity of bone-forming osteoblasts and bone-resorbing osteoclasts, ensuring the balance between bone formation and resorption. The orchestrated interplay between these cellular activities is governed by a complex network of signalling pathways, hormonal regulation, and local factors, all contributing to the maintenance of bone homeostasis [26,27]. The bone remodelling process consists of sequential phases: activation, resorption, reversal, formation, and quiescence. It typically occurs in discrete units called basic multicellular units scattered throughout the bone tissue. Each basic multicellular unit (BMU) operates independently, allowing localized remodelling without compromising the structural integrity of the entire skeleton [28]. The phases involved in the remodelling process are summarized in Table 1.

**Table 1:** The phases involved in the remodeling process The content of this table is adapted from Raggatt et al., 2010, study [29].

Phase	Description	Result
Activation Phase	Involves the recruitment of osteoclasts, stimulated by local signals, leading to the migration of osteoclast precursors to the remodeling site	Initiation of bone resorption as osteoclasts are recruited to the site.
Resorption Phase	Osteoclasts attach to the bone surface, creating an acidic microenvironment. They dissolve the mineralized matrix and release enzymes, resulting in the formation of a resorption pit.	Formation of a resorption pit through the breakdown of bone matrix, exposing collagen fibers.
Reversal Phase	It follows resorption and involves a transition from bone resorption to bone formation. Osteoblast precursors are recruited, and osteoclast activity ceases.	Shift from bone resorption to the beginning of bone formation, preparing for the next phase.
Formation Phase	Osteoblasts deposit new bone matrix within the resorption pit. Initially unmineralized, the matrix consists of collagen fibers and proteins. Over time, minerals are deposited, forming bone.	Creation of new bone as osteoblasts generate and mineralize a matrix within the resorption pit.
Quiescence Phase	After bone formation, the remodeling site enters a quiescent phase, temporarily halting cellular activities until the next remodeling cycle is initiated.	Temporary cessation of bone remodeling activities, providing a period of rest before the next cycle begins.

Role of Various Cells in Bone Remodelling

Osteoblasts are bone-forming cells derived from mesenchymal stem cells. They play a central role in bone formation by synthesizing and secreting the organic matrix of bone, which includes collagen and non-collagenous proteins. Osteoblasts also regulate mineralization by promoting the deposition of hydroxyapatite crystals [30-32]. While osteoclasts are large, multinucleated cells derived from hematopoietic stem cells. Their primary function is bone resorption. Osteoclasts attach to the bone surface, create an acidic microenvironment, and release enzymes, such as cathepsin K, that break down both the mineral and organic components of bone. Osteocytes are mature osteoblasts that have become embedded in the mineralized matrix. They play a crucial role in sensing mechanical stresses and orchestrating the remodelling process. Osteocytes communicate with other bone cells through a network of cellular processes within the bone matrix [30-32].

Signaling Pathways in Bone Remodelling

The Receptor Activator of Nuclear Factor-Kappa B (RANK), its ligand RANKL, and the decoy receptor Osteoprotegerin (OPG) form a critical signalling pathway. RANKL, produced by osteoblasts and other cells, binds to RANK on osteoclast precursors, activating them. OPG acts as a decoy receptor, preventing RANKL from binding to RANK and thus inhibiting osteoclast formation and activity. 

Wnt signalling is essential for bone formation. Activation of the Wnt pathway leads to the stabilization and nuclear translocation of β-catenin, promoting osteoblast differentiation and bone formation. Bone Morphogenetic Proteins (BMPs) are growth factors involved in bone development and maintenance. They stimulate osteoblast differentiation and bone formation, contributing to the overall balance in bone remodelling. Parathyroid Hormone (PTH) plays a crucial role in calcium homeostasis and bone remodelling. PTH stimulates bone resorption by activating osteoclasts and increases calcium reabsorption in the kidneys. However, intermittent PTH exposure can also stimulate bone formation.

Corticosteroids and bone metabolism: understanding the disruption of balance

However, a well-documented side effect of prolonged corticosteroid use is the detrimental impact on bone metabolism. To comprehend how corticosteroids contribute to bone loss, it is crucial to explore their effects on osteoblasts and osteoclasts, the two main cell types orchestrating bone formation and resorption [33].

Effects on Osteoblasts

Osteoblasts are specialized bone-forming cells crucial for the synthesis and mineralization of the bone matrix. Corticosteroids exert several effects on osteoblasts, disrupting their normal functioning and impairing bone formation (1) Inhibition of osteoblast differentiation: corticosteroids interfere with the differentiation of osteoblast precursor cells into mature osteoblasts. This inhibition reduces the pool of active bone-forming cells, compromising the ability of the bone to undergo proper remodelling and repair, (2) Suppression of collagen synthesis: corticosteroids suppress the synthesis of collagen, a key component of the bone matrix. This reduction in collagen production compromises the structural integrity of the bone, making it more susceptible to fractures, (3) Decreased osteocalcin production: osteocalcin is a protein produced by osteoblasts that plays a crucial role in bone mineralization. Corticosteroids reduce the production of osteocalcin, further impairing the process of bone mineralization and contributing to decreased bone density, (4) Induction of osteoblast apoptosis: prolonged exposure to corticosteroids induces apoptosis, or programmed cell death, in osteoblasts. This leads to fewer viable osteoblasts, impacting their ability to maintain bone homeostasis, (5) Effects on osteoclasts: osteoclasts are multinucleated cells responsible for bone resorption, a process vital for bone remodeling [33,34]. Corticosteroids influence osteoclast function, leading to increased bone resorption, (6) Enhancement of osteoclast formation: corticosteroids stimulate the formation of osteoclasts from their precursor cells. This excessive osteoclastogenesis results in an increased number of bone-resorbing cells, contributing to accelerated bone loss, (7) Prolonged osteoclast survival: corticosteroids prolong the lifespan of mature osteoclasts. Normally, osteoclasts undergo apoptosis after completing their resorption activity [35]. However, corticosteroids disrupt this natural regulatory mechanism, leading to sustained bone resorption and upregulation of RANKL (Receptor Activator of Nuclear Factor κB Ligand). Corticosteroids upregulate the expression of RANKL, a key signalling molecule that promotes osteoclast formation and activity. The increased RANKL/RANK (Receptor Activator of Nuclear Factor κB) interaction further enhances osteoclast-mediated bone resorption and disruption of the balance between bone formation and resorption. The dynamic equilibrium between bone formation by osteoblasts and bone resorption by osteoclasts is essential for maintaining bone density and strength. Corticosteroids disrupt this delicate balance, tipping the scale toward increased bone resorption and decreased bone formation, causing an imbalance in bone remodelling. Corticosteroids induce a state of heightened bone turnover, characterized by increased bone resorption and decreased bone formation. This imbalance results in a net loss of bone mass over time, contributing to the development of corticosteroid-induced osteoporosis [36], and impaired bone quality. The compromised function of osteoblasts and the excessive activity of osteoclasts lead to alterations in bone microarchitecture. Corticosteroid-induced bone loss not only reduces bone quantity but also impairs bone quality, making the skeleton more susceptible to fractures and secondary hyperparathyroidism. Corticosteroids can induce secondary hyperparathyroidism, characterized by elevated levels of parathyroid hormone (PTH). Increased PTH levels contribute to bone resorption by enhancing the activity of osteoclasts [10,37].

Factors influencing corticosteroid-induced bone loss: unraveling the complexity

Corticosteroid-induced bone loss is a significant concern for individuals undergoing prolonged corticosteroid therapy, and susceptibility to this adverse effect varies among patients. Several factors contribute to individual vulnerability, highlighting the multifaceted nature of corticosteroid-induced bone loss. Age is a pivotal factor influencing susceptibility to corticosteroid-induced bone loss. Pediatric and adolescent populations may experience more profound impacts on bone health due to the ongoing bone development and accrual during these stages. In contrast, elderly individuals already grappling with age-related bone loss may face an augmented risk. The synergy between corticosteroid-induced bone loss and age-related bone changes can result in more rapid bone deterioration [38]. Gender differences play a crucial role in the susceptibility to corticosteroid-induced bone loss. Postmenopausal women, who are already at an increased risk of osteoporosis due to hormonal changes, may be particularly vulnerable. Estrogen has a protective effect on bone density, and the decline in estrogen during menopause contributes to bone loss. The additional impact of corticosteroids further accelerates this process. However, men are not exempt, and corticosteroid-induced bone loss can affect them as well, albeit with potentially different underlying mechanisms [39]. Also, The dose and duration of corticosteroid therapy are directly correlated with the risk of bone loss. Higher doses and longer durations of exposure amplify the detrimental effects on bone metabolism. Short-term, high-dose corticosteroid therapy (e.g., for acute inflammatory conditions) may have less pronounced effects compared to chronic, low-to-moderate dose regimens. Prolonged exposure increases the cumulative impact on bone density, emphasizing the importance of carefully balancing the therapeutic benefits of corticosteroids with their potential adverse effects [40]. 

Furthermore, The underlying health condition for which corticosteroids are prescribed can significantly influence susceptibility to bone loss. Individuals with chronic inflammatory diseases, such as rheumatoid arthritis or inflammatory bowel disease, may require long-term corticosteroid therapy to manage their conditions. The inflammatory process itself can contribute to bone resorption, and when coupled with corticosteroid therapy, the risk of bone loss is heightened. Furthermore, diseases affecting calcium and vitamin D metabolism, such as renal disorders, can exacerbate corticosteroid-induced bone loss [11]. Adequate nutrition is crucial for maintaining bone health, and nutritional deficiencies can exacerbate the impact of corticosteroids on bones. Calcium and vitamin D intake is particularly important, as corticosteroids may interfere with their absorption and metabolism. Additionally, lifestyle factors such as physical inactivity and smoking contribute to decreased bone density. Addressing these modifiable risk factors through dietary interventions and lifestyle modifications can help mitigate the impact of corticosteroid-induced bone loss [41].

Genetic predisposition may influence an individual's response to corticosteroids and their impact on bone health. Polymorphisms in genes related to bone metabolism could contribute to variations in susceptibility among different individuals. Genetic factors may influence the rate of bone turnover, the efficiency of bone remodelling, and the responsiveness of osteoblasts and osteoclasts to corticosteroid-induced changes [42]. Certain medications used concomitantly with corticosteroids may either exacerbate or mitigate the risk of bone loss. For example, medications like bisphosphonates or other anti-resorptive agents may be prescribed to counteract the increased bone resorption induced by corticosteroids. On the other hand, medications such as anticonvulsants or heparin may negatively impact bone health and synergize with corticosteroids to increase the risk of osteoporosis [43].

BMD measurement: a crucial tool in assessing corticosteroid-induced bone loss

Corticosteroid-induced bone loss is a well-recognized complication of long-term corticosteroid therapy and assessing BMD plays a pivotal role in understanding and managing this adverse effect. BMD measurement provides a quantitative assessment of bone health, enabling clinicians to evaluate the impact of corticosteroids on skeletal integrity and make informed decisions regarding preventive strategies [44].

BMD measurements serve as a valuable tool for the early detection of corticosteroid-induced bone loss. By establishing baseline BMD levels before initiating corticosteroid therapy, clinicians can identify individuals at higher risk for bone-related complications. Subsequent monitoring of BMD over time allows for risk stratification, enabling early intervention in those experiencing accelerated bone loss, and quantifying the extent of bone loss. BMD measurements quantify the mineral content of bone, providing an objective measure of bone density. Changes in BMD over time reflect alterations in bone mass and can indicate the extent of corticosteroid-induced bone loss [44,45]. This information guides clinicians in assessing the severity of the condition and tailoring interventions based on the degree of bone density reduction, predicting fracture risk: low BMD is a well-established predictor of fracture risk, and BMD measurements play a crucial role in assessing the likelihood of fractures in individuals on corticosteroid therapy [44,45]. The World Health Organization (WHO) defines osteoporosis based on BMD measurements, with a T-score of -2.5 or lower indicating osteoporosis [46]. Incorporating BMD data into fracture risk assessment tools enhances the accuracy of predicting fractures in corticosteroid-treated patients, and monitoring treatment response. BMD measurements serve as a reliable indicator of treatment response in individuals undergoing interventions to mitigate corticosteroid-induced bone loss [44,45]. Monitoring changes in BMD over time allows clinicians to assess the effectiveness of preventive measures, such as lifestyle modifications, nutritional interventions, and pharmacological therapies. BMD measurements contribute to informed clinical decision-making regarding the continuation, modification, or cessation of corticosteroid therapy. The data obtained from BMD assessments guide clinicians in balancing the therapeutic benefits of corticosteroids with the associated risk of bone loss. This individualized approach is particularly relevant in chronic conditions where corticosteroids are essential for disease management [44,45].

Prevention and management strategies for corticosteroid-induced bone loss

Corticosteroid-induced bone loss is a significant concern for individuals on long-term corticosteroid therapy. Implementing effective prevention and management strategies is crucial to mitigate the adverse effects on bone health. Current recommendations emphasize a multifaceted approach, incorporating lifestyle modifications, nutritional interventions, and pharmacological treatments [47,48]. A summary of the management strategies and their potential importance is shown in Table 2.

**Table 2:** Summary of the management strategies and their potential importance

Management Strategy	Potential Importance
Calcium and Vitamin D Supplementation	Adequate intake is fundamental for maintaining bone health. Particularly vital for individuals on corticosteroid therapy to counter interference with calcium absorption and metabolism [49].
Bisphosphonates	Inhibiting bone resorption is especially recommended for individuals anticipating more than three months of corticosteroid therapy [50].
Other Antiresorptive Agents	Denosumab and SERMs like raloxifene offer alternatives to bisphosphonates. Shared goal of preserving bone density and reducing fracture risk in corticosteroid-induced bone loss [51].
Teriparatide (Parathyroid Hormone)	Approved for high fracture risk, including chronic glucocorticoid therapy cases. Stimulates bone formation as an option when other treatments are not well-tolerated or contraindicated [48].
Lifestyle Modifications	Weight-bearing exercises, smoking cessation, and limiting alcohol intake contribute to bone strength and overall health [47].
Regular Monitoring	Essential for early detection of changes in bone density. Allows adjustments to preventive or therapeutic interventions. Periodic assessments are recommended for long-term corticosteroid therapy [1].
Individualized Approach	Tailored based on factors like age, gender, health conditions, and corticosteroid regimen. Balances the benefits of therapy against potential risks to bone health [47].
Patient Education	Key to informing individuals about corticosteroid impact on bone health, lifestyle modifications, and adherence to interventions. Empowers active participation in bone health management [47].

Clinical implications of corticosteroid-induced bone loss: navigating fracture risk and patient morbidity

Corticosteroid-induced bone loss carries significant clinical implications, affecting not only bone health but also overall patient morbidity. The impact of long-term corticosteroid therapy on the skeletal system underscores the importance of close monitoring and proactive management to mitigate the associated risks [9]. One of the primary clinical implications of corticosteroid-induced bone loss is the elevated risk of fractures. Corticosteroids contribute to bone loss by disrupting the balance between bone formation and resorption, leading to decreased BMD. This reduction in BMD is a well-established risk factor for fractures. The increased fracture risk is particularly concerning for weight-bearing bones, such as the vertebrae, hips, and wrists. Fractures in these areas not only result in physical morbidity but also have significant implications for patient quality of life and functional independence [10]. Corticosteroid-induced bone loss is not uniform across all skeletal sites. Vertebrae are susceptible to fractures due to reductions in trabecular bone density. Vertebral fractures, often asymptomatic or unrecognized, can lead to progressive spinal deformities, height loss, and chronic pain. Hip fractures, associated with considerable morbidity and mortality, are another critical concern. Recognizing these site-specific vulnerabilities is crucial for tailoring preventive and management strategies to the individual patient's risk profile [37]. 

Beyond the immediate consequences of fractures, corticosteroid-induced bone loss contributes to a broader spectrum of patient morbidity. Chronic pain, functional impairment, and diminished quality of life are common outcomes. Vertebral fractures, even when asymptomatic, can lead to decreased mobility, respiratory compromise, and limitations in daily activities. Hip fractures, in addition to acute pain and immobility, are associated with increased mortality and a higher likelihood of long-term disability. Overall, the compromised bone health induced by corticosteroids significantly influences the well-being and functionality of affected individuals [9,24]. 

Close monitoring is paramount in individuals on long-term corticosteroid therapy to assess bone health and fracture risk. Regular assessments, including BMD measurements, help identify early changes in bone density and guide intervention strategies. Clinical evaluations for signs of fractures, such as back pain or height loss, are crucial for detecting vertebral fractures that may otherwise go unnoticed. Monitoring also allows healthcare providers to evaluate the effectiveness of preventive measures and adjust interventions as needed [35]. Proactive management is essential to minimize the impact of corticosteroid-induced bone loss. This includes a combination of pharmacological and non-pharmacological interventions. Bisphosphonates, such as alendronate or risedronate, are commonly prescribed to prevent and manage bone loss. Calcium and vitamin D supplementation, along with lifestyle modifications such as weight-bearing exercises and smoking cessation, contribute to comprehensive management. The choice of intervention should be individualized based on factors such as age, gender, underlying health conditions, and corticosteroid regimen [28]. Also, educating patients about the potential consequences of corticosteroid-induced bone loss and involving them in shared decision-making is crucial. Awareness empowers patients to actively participate in their bone health management, adhere to prescribed interventions, and promptly report any symptoms or concerns. Shared decision-making ensures that the chosen interventions align with the patient's preferences, values, and overall health goals [1].

## Conclusions

Corticosteroids pose serious risks to bone health and can cause osteoporosis even though they are useful in treating medical disorders. Comprehensive prevention and control measures are required due to the complex mechanisms involved, which include disruption of bone remodelling, inhibition of osteoblasts, and activation of osteoclasts. Corticosteroid-induced bone loss is complex, with individual susceptibility factors like age, gender, medication dosage, underlying medical problems, and lifestyle playing a role. It is vital to implement targeted preventive measures that are informed by site-specific vulnerabilities in bones.
